# *rac*-(1*R*,2*S*,6*R*,7*R*)-4-{[(1*E*)-(2-Chloro­phen­yl)methyl­idene]amino}-1-isopropyl-7-methyl-4-aza­tricyclo­[5.2.2.0^2,6^]undec-8-ene-3,5-dione

**DOI:** 10.1107/S1600536811013468

**Published:** 2011-04-29

**Authors:** Jian-Xin Huang, Wen-Gui Duan, Xian-Li Ma, Qi-Jin Mo, Yin-Hua Liang

**Affiliations:** a College of Chemistry & Chemical Engineering, Guangxi University, Nanning 530004, People’s Republic of China; b College of Pharmacy, Guilin Medical University, Guilin 541004, People’s Republic of China

## Abstract

The title compound, C_21_H_23_ClN_2_O_2_, was synthesized from *N*-amino-α-terpinene maleimide and 2-chloro­benzaldehyde. There are two independent mol­ecules in the asymmetric unit which are linked *via* an inter­molecular C—H⋯O hydrogen bond. The crystal studied was found to be a partial merohedral twin, with a 0.74 (7):0.26 (7) domain ratio.

## Related literature

For the synthesis of the starting α-terpinene-maleic anhydride adduct, see: Luo *et al.* (2006 ▶). For the synthesis of *N*-amino-α-terpinene maleimide, see: Maurya & Verma (1986 ▶). For related structures, see: Struga *et al.* (2007 ▶, 2009 ▶); Devarajegowda *et al.* (2010 ▶); Duan *et al.* (2007 ▶). For standard bond lengths, see: Orpen *et al.* (1989 ▶).
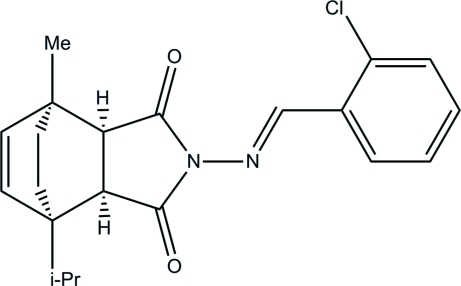


## Experimental

### 

#### Crystal data

C_21_H_23_ClN_2_O_2_
*M* *_r_* = 370.86
Orthorhombic, 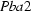

*a* = 18.505 (9) Å
*b* = 27.012 (13) Å
*c* = 7.630 (4) Å
*V* = 3814 (3) Å^3^
*Z* = 8
Mo *K*α radiation
μ = 0.22 mm^−1^
*T* = 296 K
0.35 × 0.28 × 0.25 mm

#### Data collection

Bruker SMART CCD area-detector diffractometer
Absorption correction: multi-scan (*SADABS*; Sheldrick, 1996 ▶) *T*_min_ = 0.929, *T*_max_ = 0.947
26001 measured reflections
8499 independent reflections
4632 reflections with *I* > 2σ(*I*)
*R*_int_ = 0.092

#### Refinement

*R*[*F* ^2^ > 2σ(*F* ^2^)] = 0.059
*wR*(*F* ^2^) = 0.129
*S* = 0.99
8499 reflections
470 parameters
1 restraint
H-atom parameters constrained
Δρ_max_ = 0.14 e Å^−3^
Δρ_min_ = −0.24 e Å^−3^
Absolute structure: Flack (1983 ▶), 3824 Friedel pairs
Flack parameter: 0.26 (7)

### 

Data collection: *SMART* (Bruker, 2001 ▶); cell refinement: *SAINT* (Bruker, 2002 ▶); data reduction: *SAINT*; program(s) used to solve structure: *SHELXS97* (Sheldrick, 2008 ▶); program(s) used to refine structure: *SHELXL97* (Sheldrick, 2008 ▶); molecular graphics: *SHELXTL* (Sheldrick, 2008 ▶); software used to prepare material for publication: *SHELXTL*.

## Supplementary Material

Crystal structure: contains datablocks I, global. DOI: 10.1107/S1600536811013468/ld2006sup1.cif

Structure factors: contains datablocks I. DOI: 10.1107/S1600536811013468/ld2006Isup2.hkl

Additional supplementary materials:  crystallographic information; 3D view; checkCIF report

## Figures and Tables

**Table 1:** Hydrogen-bond geometry (Å, °)

*D*—H⋯*A*	*D*—H	H⋯*A*	*D*⋯*A*	*D*—H⋯*A*
C2*B*—H2*BB*⋯O1*A*^i^	0.98	2.40	3.213 (4)	139

Symmetry code: (i) 
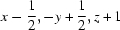
.

**Fig. 1.**
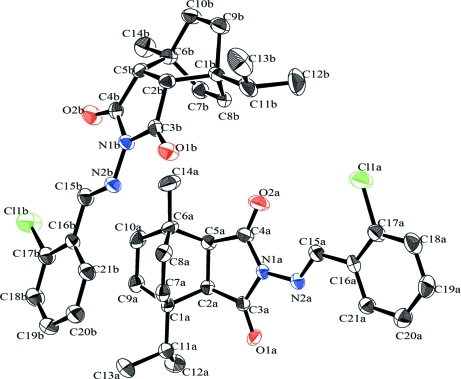
The molecular structure of the title compound with the atom numbering scheme. Displacement ellipsoids are drawn at the 30% probability level. H atoms have been omited.

## supplementary crystallographic information

### Comment

Turpentine is an abundant and green resource in China. α-Pinene, the main component of turpentine, can be isomerized to α-terpinene which reacts with maleic anhydride to yield the α-terpinene-maleic anhydride adduct (Luo *et al.*, 2006). In order to search for novel bioactive compounds (Duan *et al.*, 2007), a series of α-pinene derivatives was synthesized and their respective crystal structures were detected.

Herein, we report the crystal structure of the title compound. The structure presents racemate crystallizing in a polar space group with Flack parameter 0.26 (7). The asymmetric unit of the title compound is shown in Fig. 1. There are two independent molecules [A and B] with all bond lengths and angles within normal ranges (Orpen *et al.*, 1989). The C = N double bond in both molecules adopts E configuration. The dihedral angles between benzene rings [C16—C21 and C37—C42] and hydrazone moieties [N1A—N2A—C15A—C16A and N1B—N2B—C15B—C16B] are 4.1 (3)° and 1.7 (3)°, respectively. In the crystal, molecules A and B are linked together *via* weak intermolecular C—H···O hydrogen bond between tertiary carbon atom and carbonyl group.

### Experimental

A mixture of 3.042 g (0.015 mol) of *N*-amino-α-terpinene maleimide, 1.41 g (0.01 mol) of 2-chlorobenzaldehyde and 35 ml of ethanol was placed in a 50 ml two-necked flask. When the reaction temperature reached 84 °C, 3 ml of acetic acid was successively added to the solution as a catalyst in 15 minutes. The reaction was monitored by TLC. After complete disappearance of 2-chlorobenzaldehyde, the solvent was evaporated under reduced pressure. The residue was washed with distilled water. The crude product was purified by column chromatography and recrystallized from ethanol. Single crystals of the title compound suitable for X-ray diffraction were obtained by slow evaporation of an ethanol solution.

### Refinement

All H atoms were positioned geometrically and refined using a riding model with C–H = 0.93–0.98 Å with *U*_iso_(H) = 1.5Ueq(C) for methyl H atoms and *Uiso*(H) = 1.2*Ueq*(C) for all other H atoms. The conformation of the Me groups was optimized rotationally.

### Crystal data

**Table:**

C_21_H_23_ClN_2_O_2_	*D*_x_ = 1.292 Mg m^−^^3^
*M**_r_* = 370.86	Melting point: 409 K
Orthorhombic, *P**b**a*2	Mo *K*α radiation, λ = 0.71073 Å
Hall symbol: P 2 -2ab	Cell parameters from 5447 reflections
*a* = 18.505 (9) Å	θ = 2.3–24.8°
*b* = 27.012 (13) Å	µ = 0.22 mm^−^^1^
*c* = 7.630 (4) Å	*T* = 296 K
*V* = 3814 (3) Å^3^	Block, colourless
*Z* = 8	0.35 × 0.28 × 0.25 mm
*F*(000) = 1568	

### Data collection

**Table:**

Bruker SMART CCD area-detector diffractometer	8499 independent reflections
Radiation source: fine-focus sealed tube	4632 reflections with *I* > 2σ(*I*)
graphite	*R*_int_ = 0.092
φ and ω scans	θ_max_ = 27.4°, θ_min_ = 1.3°
Absorption correction: multi-scan (*SADABS*; Sheldrick, 1996)	*h* = −23→22
*T*_min_ = 0.929, *T*_max_ = 0.947	*k* = −34→34
26001 measured reflections	*l* = −9→9

### Refinement

**Table:**

Refinement on *F*^2^	Secondary atom site location: difference Fourier map
Least-squares matrix: full	Hydrogen site location: inferred from neighbouring sites
*R*[*F*^2^ > 2σ(*F*^2^)] = 0.059	H-atom parameters constrained
*wR*(*F*^2^) = 0.129	*w* = 1/[σ^2^(*F*_o_^2^) + (0.0308*P*)^2^] where *P* = (*F*_o_^2^ + 2*F*_c_^2^)/3
*S* = 0.99	(Δ/σ)_max_ < 0.001
8499 reflections	Δρ_max_ = 0.14 e Å^−^^3^
470 parameters	Δρ_min_ = −0.24 e Å^−^^3^
1 restraint	Absolute structure: Flack (1983), 3824 Friedel pairs
Primary atom site location: structure-invariant direct methods	Flack parameter: 0.26 (7)

### Special details

**Table:**

Geometry. All e.s.d.'s (except the e.s.d. in the dihedral angle between two l.s. planes) are estimated using the full covariance matrix. The cell e.s.d.'s are taken into account individually in the estimation of e.s.d.'s in distances, angles and torsion angles; correlations between e.s.d.'s in cell parameters are only used when they are defined by crystal symmetry. An approximate (isotropic) treatment of cell e.s.d.'s is used for estimating e.s.d.'s involving l.s. planes.
Refinement. Refinement of *F*^2^ against ALL reflections. The weighted *R*-factor *wR* and goodness of fit *S* are based on *F*^2^, conventional *R*-factors *R* are based on *F*, with *F* set to zero for negative *F*^2^. The threshold expression of *F*^2^ > σ(*F*^2^) is used only for calculating *R*-factors(gt) *etc*. and is not relevant to the choice of reflections for refinement. *R*-factors based on *F*^2^ are statistically about twice as large as those based on *F*, and *R*- factors based on ALL data will be even larger.

### Fractional atomic coordinates and isotropic or equivalent isotropic displacement parameters (Å^2^)

**Table:**

	*x*	*y*	*z*	*U*_iso_*/*U*_eq_	
Cl1A	0.84297 (7)	0.09373 (3)	0.36480 (15)	0.0834 (3)	
Cl1B	0.59584 (8)	0.44416 (3)	0.80798 (15)	0.0956 (4)	
O1A	0.91729 (14)	0.33806 (8)	0.1121 (3)	0.0617 (7)	
O2A	0.86707 (19)	0.24002 (8)	0.5824 (4)	0.0871 (10)	
O1B	0.53699 (13)	0.19579 (7)	0.5811 (3)	0.0564 (6)	
O2B	0.59021 (16)	0.29986 (8)	1.0328 (3)	0.0752 (8)	
N1A	0.88856 (15)	0.27992 (8)	0.3209 (3)	0.0434 (7)	
N2A	0.89422 (16)	0.24227 (8)	0.1979 (4)	0.0495 (7)	
N1B	0.56592 (15)	0.25645 (8)	0.7799 (3)	0.0423 (7)	
N2B	0.56132 (15)	0.29247 (8)	0.6508 (3)	0.0473 (7)	
C1A	0.82636 (18)	0.40115 (9)	0.3761 (5)	0.0409 (7)	
C2A	0.88924 (17)	0.36367 (10)	0.4107 (4)	0.0409 (8)	
H2AB	0.9341	0.3818	0.4339	0.049*	
C3A	0.90062 (18)	0.32852 (10)	0.2604 (4)	0.0432 (8)	
C4A	0.8747 (2)	0.27826 (11)	0.5006 (4)	0.0523 (9)	
C5A	0.87074 (19)	0.33034 (10)	0.5702 (4)	0.0449 (8)	
H5AA	0.9076	0.3348	0.6610	0.054*	
C6A	0.7954 (2)	0.34372 (11)	0.6468 (4)	0.0523 (9)	
C7A	0.75934 (18)	0.37079 (10)	0.3591 (5)	0.0480 (8)	
H7A	0.7302	0.3720	0.2598	0.058*	
C8A	0.7441 (2)	0.34205 (11)	0.4946 (5)	0.0525 (9)	
H8A	0.7034	0.3218	0.4955	0.063*	
C9A	0.8194 (2)	0.43166 (11)	0.5466 (5)	0.0567 (10)	
H9AA	0.8641	0.4495	0.5676	0.068*	
H9AB	0.7810	0.4558	0.5334	0.068*	
C10A	0.8030 (2)	0.39829 (11)	0.7037 (5)	0.0633 (10)	
H10A	0.7586	0.4091	0.7591	0.076*	
H10B	0.8417	0.4012	0.7890	0.076*	
C11A	0.8400 (2)	0.43375 (11)	0.2117 (5)	0.0577 (10)	
H11A	0.8408	0.4115	0.1103	0.069*	
C12A	0.9112 (2)	0.46143 (14)	0.2123 (6)	0.0878 (14)	
H12A	0.9500	0.4385	0.2314	0.132*	
H12B	0.9109	0.4856	0.3044	0.132*	
H12C	0.9178	0.4777	0.1016	0.132*	
C13A	0.7777 (3)	0.47078 (13)	0.1818 (6)	0.0898 (14)	
H13A	0.7325	0.4533	0.1808	0.135*	
H13B	0.7844	0.4873	0.0715	0.135*	
H13C	0.7774	0.4948	0.2745	0.135*	
C14A	0.7731 (3)	0.31109 (13)	0.7997 (5)	0.0815 (13)	
H14A	0.7693	0.2774	0.7609	0.122*	
H14B	0.7272	0.3220	0.8440	0.122*	
H14C	0.8087	0.3133	0.8909	0.122*	
C15A	0.88097 (19)	0.19845 (10)	0.2429 (5)	0.0531 (9)	
H15A	0.8705	0.1909	0.3591	0.064*	
C16A	0.88219 (17)	0.15936 (10)	0.1101 (4)	0.0442 (8)	
C17A	0.86575 (19)	0.11071 (11)	0.1516 (5)	0.0547 (9)	
C18A	0.8649 (2)	0.07424 (12)	0.0247 (6)	0.0676 (11)	
H18A	0.8531	0.0419	0.0549	0.081*	
C19A	0.8815 (2)	0.08581 (13)	−0.1468 (6)	0.0726 (12)	
H19A	0.8819	0.0611	−0.2317	0.087*	
C20A	0.8977 (2)	0.13413 (12)	−0.1928 (5)	0.0618 (10)	
H20A	0.9081	0.1422	−0.3086	0.074*	
C21A	0.89824 (18)	0.17020 (12)	−0.0641 (4)	0.0497 (9)	
H21A	0.9096	0.2026	−0.0947	0.060*	
C1B	0.62464 (19)	0.13513 (10)	0.8637 (4)	0.0468 (8)	
C2B	0.56289 (17)	0.17445 (9)	0.8870 (4)	0.0410 (8)	
H2BB	0.5174	0.1573	0.9118	0.049*	
C3B	0.55280 (17)	0.20729 (10)	0.7289 (4)	0.0411 (8)	
C4B	0.58056 (19)	0.26101 (11)	0.9590 (4)	0.0487 (9)	
C5B	0.58019 (19)	0.20992 (10)	1.0396 (4)	0.0426 (8)	
H5BA	0.5415	0.2080	1.1271	0.051*	
C6B	0.65302 (19)	0.19637 (11)	1.1273 (4)	0.0484 (9)	
C7B	0.70786 (19)	0.19481 (12)	0.9834 (5)	0.0536 (9)	
H7B	0.7496	0.2140	0.9862	0.064*	
C8B	0.69336 (18)	0.16448 (10)	0.8521 (5)	0.0489 (9)	
H8B	0.7243	0.1615	0.7567	0.059*	
C9B	0.6263 (2)	0.10767 (11)	1.0426 (5)	0.0593 (10)	
H9BA	0.5801	0.0916	1.0619	0.071*	
H9BB	0.6632	0.0822	1.0391	0.071*	
C10B	0.6420 (2)	0.14294 (11)	1.1955 (5)	0.0584 (10)	
H10C	0.6851	0.1321	1.2568	0.070*	
H10D	0.6020	0.1423	1.2776	0.070*	
C11B	0.6128 (2)	0.10039 (11)	0.7077 (5)	0.0674 (11)	
H11B	0.6118	0.1211	0.6023	0.081*	
C12B	0.6761 (3)	0.06402 (14)	0.6857 (7)	0.0962 (16)	
H12D	0.7207	0.0821	0.6822	0.144*	
H12E	0.6703	0.0459	0.5784	0.144*	
H12F	0.6768	0.0414	0.7826	0.144*	
C13B	0.5421 (3)	0.07234 (13)	0.7127 (6)	0.0925 (15)	
H13D	0.5028	0.0953	0.7239	0.139*	
H13E	0.5420	0.0502	0.8110	0.139*	
H13F	0.5365	0.0537	0.6064	0.139*	
C14B	0.6738 (2)	0.23070 (13)	1.2780 (5)	0.0724 (11)	
H14D	0.6803	0.2637	1.2342	0.109*	
H14E	0.7180	0.2194	1.3302	0.109*	
H14F	0.6361	0.2306	1.3645	0.109*	
C15B	0.56802 (18)	0.33766 (10)	0.6934 (5)	0.0512 (9)	
H15B	0.5747	0.3467	0.8100	0.061*	
C16B	0.56511 (18)	0.37540 (10)	0.5557 (4)	0.0436 (8)	
C17B	0.5779 (2)	0.42523 (11)	0.5944 (5)	0.0559 (10)	
C18B	0.5762 (2)	0.46066 (12)	0.4637 (5)	0.0678 (12)	
H18B	0.5843	0.4938	0.4910	0.081*	
C19B	0.5628 (2)	0.44701 (13)	0.2937 (6)	0.0689 (11)	
H19B	0.5618	0.4709	0.2059	0.083*	
C20B	0.5508 (2)	0.39830 (12)	0.2528 (5)	0.0626 (10)	
H20B	0.5421	0.3891	0.1373	0.075*	
C21B	0.55172 (19)	0.36324 (10)	0.3818 (5)	0.0536 (9)	
H21B	0.5431	0.3303	0.3524	0.064*	

### Atomic displacement parameters (Å^2^)

**Table:**

	*U*^11^	*U*^22^	*U*^33^	*U*^12^	*U*^13^	*U*^23^
Cl1A	0.1296 (10)	0.0559 (5)	0.0648 (6)	−0.0107 (5)	0.0002 (7)	0.0126 (5)
Cl1B	0.1695 (13)	0.0502 (5)	0.0670 (7)	−0.0153 (6)	−0.0046 (8)	−0.0105 (5)
O1A	0.0818 (19)	0.0451 (12)	0.0583 (16)	0.0061 (11)	0.0360 (15)	0.0021 (11)
O2A	0.162 (3)	0.0432 (13)	0.0559 (16)	0.0018 (15)	0.0060 (18)	0.0129 (13)
O1B	0.0789 (19)	0.0427 (11)	0.0476 (14)	−0.0058 (11)	−0.0128 (14)	0.0002 (11)
O2B	0.129 (3)	0.0408 (12)	0.0555 (15)	0.0054 (13)	−0.0069 (16)	−0.0111 (12)
N1A	0.0562 (19)	0.0316 (12)	0.0425 (16)	0.0054 (11)	0.0005 (14)	−0.0027 (11)
N2A	0.068 (2)	0.0327 (13)	0.0480 (16)	0.0056 (12)	0.0024 (15)	−0.0030 (12)
N1B	0.0549 (19)	0.0315 (12)	0.0405 (15)	0.0001 (11)	−0.0014 (14)	−0.0003 (12)
N2B	0.061 (2)	0.0350 (13)	0.0462 (16)	0.0038 (12)	0.0009 (15)	0.0033 (12)
C1A	0.050 (2)	0.0336 (13)	0.0393 (16)	0.0017 (13)	0.0069 (17)	−0.0045 (14)
C2A	0.040 (2)	0.0358 (15)	0.0465 (19)	−0.0050 (13)	0.0001 (16)	−0.0020 (14)
C3A	0.042 (2)	0.0384 (15)	0.049 (2)	0.0016 (14)	0.0073 (18)	0.0030 (15)
C4A	0.072 (3)	0.0415 (18)	0.044 (2)	−0.0013 (16)	−0.0072 (19)	0.0032 (16)
C5A	0.054 (2)	0.0422 (16)	0.0387 (18)	−0.0005 (14)	−0.0072 (17)	−0.0029 (15)
C6A	0.070 (3)	0.0517 (18)	0.0355 (19)	−0.0083 (16)	0.0093 (19)	−0.0014 (15)
C7A	0.047 (2)	0.0475 (16)	0.049 (2)	0.0065 (15)	−0.0037 (19)	−0.0114 (17)
C8A	0.052 (3)	0.0486 (18)	0.057 (2)	−0.0080 (16)	0.0038 (19)	−0.0088 (17)
C9A	0.070 (3)	0.0426 (17)	0.057 (2)	−0.0029 (16)	0.001 (2)	−0.0142 (17)
C10A	0.081 (3)	0.059 (2)	0.049 (2)	−0.0013 (19)	0.009 (2)	−0.0191 (19)
C11A	0.078 (3)	0.0412 (17)	0.054 (2)	0.0074 (17)	0.010 (2)	0.0014 (16)
C12A	0.102 (4)	0.062 (2)	0.100 (3)	−0.011 (2)	0.030 (3)	0.022 (2)
C13A	0.122 (4)	0.055 (2)	0.093 (3)	0.029 (2)	0.009 (3)	0.019 (2)
C14A	0.107 (4)	0.080 (2)	0.058 (2)	−0.019 (2)	0.024 (3)	0.007 (2)
C15A	0.066 (3)	0.0406 (17)	0.053 (2)	0.0062 (16)	−0.0008 (19)	0.0020 (16)
C16A	0.043 (2)	0.0335 (16)	0.056 (2)	0.0059 (13)	−0.0043 (18)	−0.0038 (15)
C17A	0.065 (3)	0.0424 (18)	0.057 (2)	0.0004 (16)	−0.005 (2)	0.0041 (17)
C18A	0.088 (3)	0.0380 (17)	0.077 (3)	0.0016 (18)	−0.009 (3)	−0.007 (2)
C19A	0.096 (3)	0.054 (2)	0.068 (3)	0.007 (2)	0.000 (3)	−0.024 (2)
C20A	0.068 (3)	0.061 (2)	0.057 (2)	0.0008 (18)	0.002 (2)	−0.007 (2)
C21A	0.054 (3)	0.0448 (17)	0.051 (2)	−0.0027 (16)	0.0012 (18)	0.0011 (16)
C1B	0.065 (2)	0.0341 (14)	0.0419 (18)	0.0041 (15)	0.0024 (19)	0.0068 (14)
C2B	0.050 (2)	0.0329 (13)	0.0402 (18)	−0.0082 (13)	0.0024 (17)	0.0014 (14)
C3B	0.037 (2)	0.0365 (15)	0.049 (2)	0.0015 (13)	0.0015 (17)	0.0000 (15)
C4B	0.061 (3)	0.0371 (17)	0.049 (2)	0.0054 (15)	0.0076 (19)	−0.0071 (15)
C5B	0.049 (2)	0.0419 (15)	0.0373 (17)	−0.0034 (14)	0.0114 (17)	−0.0009 (14)
C6B	0.055 (2)	0.0458 (17)	0.044 (2)	−0.0056 (15)	−0.0006 (19)	0.0026 (15)
C7B	0.049 (2)	0.0490 (18)	0.063 (2)	−0.0007 (16)	0.001 (2)	0.0039 (17)
C8B	0.049 (2)	0.0455 (16)	0.052 (2)	0.0128 (15)	0.0141 (19)	0.0092 (16)
C9B	0.079 (3)	0.0420 (17)	0.057 (2)	−0.0041 (17)	0.000 (2)	0.0095 (17)
C10B	0.076 (3)	0.0540 (19)	0.045 (2)	−0.0007 (17)	−0.003 (2)	0.0128 (17)
C11B	0.111 (3)	0.0342 (16)	0.056 (2)	0.010 (2)	−0.004 (2)	−0.0030 (16)
C12B	0.135 (4)	0.060 (2)	0.094 (3)	0.033 (2)	0.000 (3)	−0.024 (2)
C13B	0.120 (4)	0.056 (2)	0.102 (4)	−0.005 (2)	−0.045 (3)	−0.018 (2)
C14B	0.090 (3)	0.073 (2)	0.054 (2)	−0.009 (2)	−0.015 (2)	−0.011 (2)
C15B	0.061 (2)	0.0382 (16)	0.054 (2)	0.0017 (15)	0.000 (2)	−0.0045 (16)
C16B	0.046 (2)	0.0350 (15)	0.050 (2)	0.0053 (13)	0.0028 (18)	0.0049 (15)
C17B	0.072 (3)	0.0391 (17)	0.057 (2)	−0.0002 (16)	0.002 (2)	−0.0033 (16)
C18B	0.100 (3)	0.0320 (17)	0.072 (3)	0.0002 (18)	0.007 (3)	0.0018 (18)
C19B	0.090 (3)	0.0492 (19)	0.068 (3)	0.0096 (19)	0.013 (3)	0.0180 (19)
C20B	0.079 (3)	0.054 (2)	0.055 (2)	0.0103 (19)	0.001 (2)	0.0018 (17)
C21B	0.065 (3)	0.0364 (15)	0.059 (2)	0.0035 (15)	0.000 (2)	0.0042 (17)

### Geometric parameters (Å, °)

**Table:**

Cl1A—C17A	1.742 (4)	C18A—C19A	1.381 (6)
Cl1B—C17B	1.740 (4)	C18A—H18A	0.9300
O1A—C3A	1.201 (4)	C19A—C20A	1.384 (5)
O2A—C4A	1.215 (4)	C19A—H19A	0.9300
O1B—C3B	1.205 (4)	C20A—C21A	1.384 (4)
O2B—C4B	1.204 (4)	C20A—H20A	0.9300
N1A—N2A	1.388 (3)	C21A—H21A	0.9300
N1A—C4A	1.396 (4)	C1B—C8B	1.501 (4)
N1A—C3A	1.409 (4)	C1B—C11B	1.532 (5)
N2A—C15A	1.257 (4)	C1B—C9B	1.554 (5)
N1B—N2B	1.388 (3)	C1B—C2B	1.570 (4)
N1B—C4B	1.398 (4)	C2B—C3B	1.509 (4)
N1B—C3B	1.405 (4)	C2B—C5B	1.542 (4)
N2B—C15B	1.269 (3)	C2B—H2BB	0.9800
C1A—C7A	1.492 (4)	C4B—C5B	1.511 (4)
C1A—C9A	1.546 (4)	C5B—C6B	1.548 (5)
C1A—C11A	1.553 (5)	C5B—H5BA	0.9800
C1A—C2A	1.565 (4)	C6B—C7B	1.495 (5)
C2A—C3A	1.504 (4)	C6B—C14B	1.527 (4)
C2A—C5A	1.552 (4)	C6B—C10B	1.548 (4)
C2A—H2AB	0.9800	C7B—C8B	1.322 (4)
C4A—C5A	1.506 (4)	C7B—H7B	0.9300
C5A—C6A	1.554 (5)	C8B—H8B	0.9300
C5A—H5AA	0.9800	C9B—C10B	1.534 (5)
C6A—C8A	1.502 (5)	C9B—H9BA	0.9700
C6A—C14A	1.519 (5)	C9B—H9BB	0.9700
C6A—C10A	1.543 (4)	C10B—H10C	0.9700
C7A—C8A	1.324 (4)	C10B—H10D	0.9700
C7A—H7A	0.9300	C11B—C13B	1.513 (6)
C8A—H8A	0.9300	C11B—C12B	1.538 (5)
C9A—C10A	1.530 (5)	C11B—H11B	0.9800
C9A—H9AA	0.9700	C12B—H12D	0.9600
C9A—H9AB	0.9700	C12B—H12E	0.9600
C10A—H10A	0.9700	C12B—H12F	0.9600
C10A—H10B	0.9700	C13B—H13D	0.9600
C11A—C12A	1.515 (5)	C13B—H13E	0.9600
C11A—C13A	1.543 (5)	C13B—H13F	0.9600
C11A—H11A	0.9800	C14B—H14D	0.9600
C12A—H12A	0.9600	C14B—H14E	0.9600
C12A—H12B	0.9600	C14B—H14F	0.9600
C12A—H12C	0.9600	C15B—C16B	1.465 (4)
C13A—H13A	0.9600	C15B—H15B	0.9300
C13A—H13B	0.9600	C16B—C21B	1.389 (5)
C13A—H13C	0.9600	C16B—C17B	1.398 (4)
C14A—H14A	0.9600	C17B—C18B	1.383 (5)
C14A—H14B	0.9600	C18B—C19B	1.371 (5)
C14A—H14C	0.9600	C18B—H18B	0.9300
C15A—C16A	1.463 (4)	C19B—C20B	1.370 (5)
C15A—H15A	0.9300	C19B—H19B	0.9300
C16A—C17A	1.385 (4)	C20B—C21B	1.366 (5)
C16A—C21A	1.393 (5)	C20B—H20B	0.9300
C17A—C18A	1.382 (5)	C21B—H21B	0.9300
			
N2A—N1A—C4A	130.9 (2)	C19A—C20A—H20A	120.5
N2A—N1A—C3A	116.7 (3)	C20A—C21A—C16A	121.9 (3)
C4A—N1A—C3A	112.4 (3)	C20A—C21A—H21A	119.1
C15A—N2A—N1A	119.4 (3)	C16A—C21A—H21A	119.1
N2B—N1B—C4B	130.1 (2)	C8B—C1B—C11B	113.5 (3)
N2B—N1B—C3B	117.1 (2)	C8B—C1B—C9B	106.7 (3)
C4B—N1B—C3B	112.8 (3)	C11B—C1B—C9B	113.2 (2)
C15B—N2B—N1B	119.1 (3)	C8B—C1B—C2B	105.4 (2)
C7A—C1A—C9A	107.3 (3)	C11B—C1B—C2B	113.5 (3)
C7A—C1A—C11A	112.1 (3)	C9B—C1B—C2B	103.8 (3)
C9A—C1A—C11A	113.0 (2)	C3B—C2B—C5B	105.3 (2)
C7A—C1A—C2A	106.1 (2)	C3B—C2B—C1B	113.4 (3)
C9A—C1A—C2A	105.4 (3)	C5B—C2B—C1B	110.8 (3)
C11A—C1A—C2A	112.5 (3)	C3B—C2B—H2BB	109.1
C3A—C2A—C5A	105.2 (2)	C5B—C2B—H2BB	109.1
C3A—C2A—C1A	112.6 (3)	C1B—C2B—H2BB	109.1
C5A—C2A—C1A	110.1 (3)	O1B—C3B—N1B	123.0 (3)
C3A—C2A—H2AB	109.6	O1B—C3B—C2B	128.8 (3)
C5A—C2A—H2AB	109.6	N1B—C3B—C2B	108.2 (3)
C1A—C2A—H2AB	109.6	O2B—C4B—N1B	124.2 (3)
O1A—C3A—N1A	123.3 (3)	O2B—C4B—C5B	127.3 (3)
O1A—C3A—C2A	128.2 (3)	N1B—C4B—C5B	108.5 (3)
N1A—C3A—C2A	108.4 (3)	C4B—C5B—C2B	105.1 (3)
O2A—C4A—N1A	123.6 (3)	C4B—C5B—C6B	112.8 (3)
O2A—C4A—C5A	127.4 (3)	C2B—C5B—C6B	111.1 (2)
N1A—C4A—C5A	109.0 (3)	C4B—C5B—H5BA	109.2
C4A—C5A—C2A	104.8 (3)	C2B—C5B—H5BA	109.2
C4A—C5A—C6A	113.2 (3)	C6B—C5B—H5BA	109.2
C2A—C5A—C6A	111.0 (2)	C7B—C6B—C14B	113.5 (3)
C4A—C5A—H5AA	109.3	C7B—C6B—C10B	108.1 (3)
C2A—C5A—H5AA	109.3	C14B—C6B—C10B	110.2 (3)
C6A—C5A—H5AA	109.3	C7B—C6B—C5B	106.3 (3)
C8A—C6A—C14A	113.9 (3)	C14B—C6B—C5B	113.6 (3)
C8A—C6A—C10A	107.7 (3)	C10B—C6B—C5B	104.5 (3)
C14A—C6A—C10A	111.3 (3)	C8B—C7B—C6B	115.9 (3)
C8A—C6A—C5A	105.6 (3)	C8B—C7B—H7B	122.1
C14A—C6A—C5A	113.5 (3)	C6B—C7B—H7B	122.1
C10A—C6A—C5A	104.3 (3)	C7B—C8B—C1B	117.0 (3)
C8A—C7A—C1A	115.6 (3)	C7B—C8B—H8B	121.5
C8A—C7A—H7A	122.2	C1B—C8B—H8B	121.5
C1A—C7A—H7A	122.2	C10B—C9B—C1B	112.1 (2)
C7A—C8A—C6A	116.8 (3)	C10B—C9B—H9BA	109.2
C7A—C8A—H8A	121.6	C1B—C9B—H9BA	109.2
C6A—C8A—H8A	121.6	C10B—C9B—H9BB	109.2
C10A—C9A—C1A	111.2 (2)	C1B—C9B—H9BB	109.2
C10A—C9A—H9AA	109.4	H9BA—C9B—H9BB	107.9
C1A—C9A—H9AA	109.4	C9B—C10B—C6B	110.4 (3)
C10A—C9A—H9AB	109.4	C9B—C10B—H10C	109.6
C1A—C9A—H9AB	109.4	C6B—C10B—H10C	109.6
H9AA—C9A—H9AB	108.0	C9B—C10B—H10D	109.6
C9A—C10A—C6A	111.1 (3)	C6B—C10B—H10D	109.6
C9A—C10A—H10A	109.4	H10C—C10B—H10D	108.1
C6A—C10A—H10A	109.4	C13B—C11B—C1B	114.3 (4)
C9A—C10A—H10B	109.4	C13B—C11B—C12B	110.0 (3)
C6A—C10A—H10B	109.4	C1B—C11B—C12B	111.6 (3)
H10A—C10A—H10B	108.0	C13B—C11B—H11B	106.9
C12A—C11A—C13A	109.3 (3)	C1B—C11B—H11B	106.9
C12A—C11A—C1A	114.7 (3)	C12B—C11B—H11B	106.9
C13A—C11A—C1A	111.4 (3)	C11B—C12B—H12D	109.5
C12A—C11A—H11A	107.0	C11B—C12B—H12E	109.5
C13A—C11A—H11A	107.0	H12D—C12B—H12E	109.5
C1A—C11A—H11A	107.0	C11B—C12B—H12F	109.5
C11A—C12A—H12A	109.5	H12D—C12B—H12F	109.5
C11A—C12A—H12B	109.5	H12E—C12B—H12F	109.5
H12A—C12A—H12B	109.5	C11B—C13B—H13D	109.5
C11A—C12A—H12C	109.5	C11B—C13B—H13E	109.5
H12A—C12A—H12C	109.5	H13D—C13B—H13E	109.5
H12B—C12A—H12C	109.5	C11B—C13B—H13F	109.5
C11A—C13A—H13A	109.5	H13D—C13B—H13F	109.5
C11A—C13A—H13B	109.5	H13E—C13B—H13F	109.5
H13A—C13A—H13B	109.5	C6B—C14B—H14D	109.5
C11A—C13A—H13C	109.5	C6B—C14B—H14E	109.5
H13A—C13A—H13C	109.5	H14D—C14B—H14E	109.5
H13B—C13A—H13C	109.5	C6B—C14B—H14F	109.5
C6A—C14A—H14A	109.5	H14D—C14B—H14F	109.5
C6A—C14A—H14B	109.5	H14E—C14B—H14F	109.5
H14A—C14A—H14B	109.5	N2B—C15B—C16B	118.8 (3)
C6A—C14A—H14C	109.5	N2B—C15B—H15B	120.6
H14A—C14A—H14C	109.5	C16B—C15B—H15B	120.6
H14B—C14A—H14C	109.5	C21B—C16B—C17B	117.4 (3)
N2A—C15A—C16A	119.2 (3)	C21B—C16B—C15B	121.8 (3)
N2A—C15A—H15A	120.4	C17B—C16B—C15B	120.8 (3)
C16A—C15A—H15A	120.4	C18B—C17B—C16B	120.7 (3)
C17A—C16A—C21A	117.7 (3)	C18B—C17B—Cl1B	118.5 (3)
C17A—C16A—C15A	121.5 (3)	C16B—C17B—Cl1B	120.9 (3)
C21A—C16A—C15A	120.8 (3)	C19B—C18B—C17B	120.0 (3)
C18A—C17A—C16A	121.2 (3)	C19B—C18B—H18B	120.0
C18A—C17A—Cl1A	117.6 (3)	C17B—C18B—H18B	120.0
C16A—C17A—Cl1A	121.1 (3)	C20B—C19B—C18B	120.2 (3)
C19A—C18A—C17A	120.0 (3)	C20B—C19B—H19B	119.9
C19A—C18A—H18A	120.0	C18B—C19B—H19B	119.9
C17A—C18A—H18A	120.0	C21B—C20B—C19B	120.0 (4)
C18A—C19A—C20A	120.1 (3)	C21B—C20B—H20B	120.0
C18A—C19A—H19A	119.9	C19B—C20B—H20B	120.0
C20A—C19A—H19A	119.9	C20B—C21B—C16B	121.8 (3)
C21A—C20A—C19A	119.0 (4)	C20B—C21B—H21B	119.1
C21A—C20A—H20A	120.5	C16B—C21B—H21B	119.1
			
C4A—N1A—N2A—C15A	5.3 (5)	C17A—C16A—C21A—C20A	0.2 (5)
C3A—N1A—N2A—C15A	−177.0 (3)	C15A—C16A—C21A—C20A	−178.2 (3)
C4B—N1B—N2B—C15B	−3.2 (5)	C8B—C1B—C2B—C3B	66.1 (3)
C3B—N1B—N2B—C15B	175.4 (3)	C11B—C1B—C2B—C3B	−58.7 (3)
C7A—C1A—C2A—C3A	63.0 (3)	C9B—C1B—C2B—C3B	178.1 (3)
C9A—C1A—C2A—C3A	176.5 (2)	C8B—C1B—C2B—C5B	−52.1 (3)
C11A—C1A—C2A—C3A	−59.9 (3)	C11B—C1B—C2B—C5B	−176.9 (3)
C7A—C1A—C2A—C5A	−54.1 (3)	C9B—C1B—C2B—C5B	59.9 (3)
C9A—C1A—C2A—C5A	59.5 (3)	N2B—N1B—C3B—O1B	−1.2 (5)
C11A—C1A—C2A—C5A	−177.0 (3)	C4B—N1B—C3B—O1B	177.7 (3)
N2A—N1A—C3A—O1A	−2.3 (5)	N2B—N1B—C3B—C2B	178.2 (2)
C4A—N1A—C3A—O1A	175.9 (3)	C4B—N1B—C3B—C2B	−2.9 (4)
N2A—N1A—C3A—C2A	177.9 (2)	C5B—C2B—C3B—O1B	−178.2 (3)
C4A—N1A—C3A—C2A	−4.0 (4)	C1B—C2B—C3B—O1B	60.5 (4)
C5A—C2A—C3A—O1A	−178.5 (4)	C5B—C2B—C3B—N1B	2.4 (3)
C1A—C2A—C3A—O1A	61.6 (5)	C1B—C2B—C3B—N1B	−118.9 (3)
C5A—C2A—C3A—N1A	1.4 (3)	N2B—N1B—C4B—O2B	2.1 (6)
C1A—C2A—C3A—N1A	−118.6 (3)	C3B—N1B—C4B—O2B	−176.6 (3)
N2A—N1A—C4A—O2A	2.4 (6)	N2B—N1B—C4B—C5B	−179.2 (3)
C3A—N1A—C4A—O2A	−175.5 (3)	C3B—N1B—C4B—C5B	2.1 (4)
N2A—N1A—C4A—C5A	−177.2 (3)	O2B—C4B—C5B—C2B	178.2 (3)
C3A—N1A—C4A—C5A	5.0 (4)	N1B—C4B—C5B—C2B	−0.5 (3)
O2A—C4A—C5A—C2A	176.7 (4)	O2B—C4B—C5B—C6B	−60.6 (4)
N1A—C4A—C5A—C2A	−3.8 (4)	N1B—C4B—C5B—C6B	120.7 (3)
O2A—C4A—C5A—C6A	−62.3 (5)	C3B—C2B—C5B—C4B	−1.2 (3)
N1A—C4A—C5A—C6A	117.3 (3)	C1B—C2B—C5B—C4B	121.8 (3)
C3A—C2A—C5A—C4A	1.4 (3)	C3B—C2B—C5B—C6B	−123.5 (3)
C1A—C2A—C5A—C4A	123.0 (3)	C1B—C2B—C5B—C6B	−0.5 (3)
C3A—C2A—C5A—C6A	−121.1 (3)	C4B—C5B—C6B—C7B	−64.2 (3)
C1A—C2A—C5A—C6A	0.5 (3)	C2B—C5B—C6B—C7B	53.5 (3)
C4A—C5A—C6A—C8A	−64.9 (3)	C4B—C5B—C6B—C14B	61.4 (4)
C2A—C5A—C6A—C8A	52.6 (3)	C2B—C5B—C6B—C14B	179.1 (3)
C4A—C5A—C6A—C14A	60.5 (4)	C4B—C5B—C6B—C10B	−178.4 (3)
C2A—C5A—C6A—C14A	178.0 (3)	C2B—C5B—C6B—C10B	−60.6 (3)
C4A—C5A—C6A—C10A	−178.3 (3)	C14B—C6B—C7B—C8B	178.1 (3)
C2A—C5A—C6A—C10A	−60.8 (3)	C10B—C6B—C7B—C8B	55.5 (4)
C9A—C1A—C7A—C8A	−55.3 (3)	C5B—C6B—C7B—C8B	−56.2 (3)
C11A—C1A—C7A—C8A	−179.9 (3)	C6B—C7B—C8B—C1B	−0.6 (4)
C2A—C1A—C7A—C8A	57.0 (4)	C11B—C1B—C8B—C7B	−179.0 (3)
C1A—C7A—C8A—C6A	0.2 (4)	C9B—C1B—C8B—C7B	−53.7 (3)
C14A—C6A—C8A—C7A	178.1 (3)	C2B—C1B—C8B—C7B	56.2 (3)
C10A—C6A—C8A—C7A	54.2 (4)	C8B—C1B—C9B—C10B	51.6 (4)
C5A—C6A—C8A—C7A	−56.8 (4)	C11B—C1B—C9B—C10B	177.1 (3)
C7A—C1A—C9A—C10A	53.7 (4)	C2B—C1B—C9B—C10B	−59.4 (3)
C11A—C1A—C9A—C10A	177.7 (3)	C1B—C9B—C10B—C6B	−0.9 (4)
C2A—C1A—C9A—C10A	−59.1 (3)	C7B—C6B—C10B—C9B	−51.5 (4)
C1A—C9A—C10A—C6A	−2.0 (4)	C14B—C6B—C10B—C9B	−176.1 (3)
C8A—C6A—C10A—C9A	−49.9 (4)	C5B—C6B—C10B—C9B	61.4 (4)
C14A—C6A—C10A—C9A	−175.3 (3)	C8B—C1B—C11B—C13B	−177.2 (3)
C5A—C6A—C10A—C9A	62.0 (4)	C9B—C1B—C11B—C13B	61.1 (4)
C7A—C1A—C11A—C12A	−173.9 (3)	C2B—C1B—C11B—C13B	−56.8 (4)
C9A—C1A—C11A—C12A	64.7 (4)	C8B—C1B—C11B—C12B	57.3 (4)
C2A—C1A—C11A—C12A	−54.5 (4)	C9B—C1B—C11B—C12B	−64.5 (4)
C7A—C1A—C11A—C13A	61.2 (4)	C2B—C1B—C11B—C12B	177.6 (3)
C9A—C1A—C11A—C13A	−60.1 (4)	N1B—N2B—C15B—C16B	178.3 (3)
C2A—C1A—C11A—C13A	−179.3 (3)	N2B—C15B—C16B—C21B	3.2 (5)
N1A—N2A—C15A—C16A	175.9 (3)	N2B—C15B—C16B—C17B	−174.9 (3)
N2A—C15A—C16A—C17A	−177.9 (3)	C21B—C16B—C17B—C18B	0.8 (5)
N2A—C15A—C16A—C21A	0.4 (5)	C15B—C16B—C17B—C18B	179.0 (3)
C21A—C16A—C17A—C18A	−0.3 (5)	C21B—C16B—C17B—Cl1B	−179.5 (3)
C15A—C16A—C17A—C18A	178.1 (3)	C15B—C16B—C17B—Cl1B	−1.3 (5)
C21A—C16A—C17A—Cl1A	−178.5 (3)	C16B—C17B—C18B—C19B	−0.8 (6)
C15A—C16A—C17A—Cl1A	−0.2 (5)	Cl1B—C17B—C18B—C19B	179.5 (4)
C16A—C17A—C18A—C19A	0.9 (6)	C17B—C18B—C19B—C20B	0.1 (6)
Cl1A—C17A—C18A—C19A	179.1 (3)	C18B—C19B—C20B—C21B	0.5 (6)
C17A—C18A—C19A—C20A	−1.3 (6)	C19B—C20B—C21B—C16B	−0.5 (6)
C18A—C19A—C20A—C21A	1.2 (6)	C17B—C16B—C21B—C20B	−0.1 (5)
C19A—C20A—C21A—C16A	−0.7 (6)	C15B—C16B—C21B—C20B	−178.3 (3)

### Hydrogen-bond geometry (Å, °)

**Table:**

*D*—H···*A*	*D*—H	H···*A*	*D*···*A*	*D*—H···*A*
C2B—H2BB···O1A^i^	0.98	2.40	3.213 (4)	139

Symmetry codes: (i) *x*−1/2, −*y*+1/2, *z*+1.
